# The Soundscapes of Lakes across an Urbanization Gradient

**DOI:** 10.1371/journal.pone.0055661

**Published:** 2013-02-12

**Authors:** Lauren M. Kuehne, Britta L. Padgham, Julian D. Olden

**Affiliations:** 1 School of Aquatic and Fishery Sciences, University of Washington, Seattle, Washington, United States of America; 2 School of Marine and Environmental Affairs, University of Washington, Seattle, Washington, United States of America; Pacific Northwest National Laboratory, United States of America

## Abstract

**Background/Methodology:**

A significant implication of increasing urbanization is anthropogenic noise pollution. Although noise is strongly associated with disruption of animal communication systems and negative health effects for humans, the study of these consequences at ecologically relevant spatial and temporal scales (termed soundscape ecology) is in early stages of application. In this study, we examined the above- and below-water soundscape of recreational and residential lakes in the region surrounding a large metropolitan area. Using univariate and multivariate approaches we test the importance of large- and local-scale landscape factors in driving acoustic characteristics across an urbanization gradient, and visualize changes in the soundscape over space and time.

**Principal Findings:**

Anthropogenic noise (anthrophony) was strongly predicted by a landcover-based metric of urbanization (within a 10 km radius), with presence of a public park as a secondary influence; this urbanization signal was apparent even in below-water recordings. The percent of hourly measurements exceeding noise thresholds associated with outdoor disturbance was 67%, 17%, and 0%, respectively, for lakes characterized as High, Medium, and Low urbanization. Decreased biophony (proportion of natural sounds) was associated with presence of a public park followed by increased urbanization; time of day was also a significant predictor of biophony. Local-scale (shoreline) residential development was not related to changes in anthrophony or biophony. The patterns we identify are illustrated with a multivariate approach which allows use of entire sound samples and facilitates interpretation of changes in a soundscape.

**Conclusions/Significance:**

As highly valued residential and recreation areas, lakes represent everyday soundscapes important to both humans and wildlife. Our findings that many of these areas, particularly those with public parks, routinely experience sound types and levels associated with disturbance, suggests that urban planners need to account for the effect of increasing development on soundscapes to avoid compromising goals for ecological and human health.

## Introduction

Rates of population growth, development, and resource extraction have drastically altered landscapes around the globe [1], [2]. The increased conversion of natural spaces to rural, suburban, or urbanized environments as well as transport of goods and people comes with increased costs of environmental noise pollution [3]. In undeveloped or protected natural areas, where lack of access may preclude other forms of landscape alteration, noise pollution remains a primary environmental threat [4], [5]. As with many common-pool environmental resources, efforts to mange environmental noise pollution are challenged by the need to balance competing demands of multiple users, difficulty or cost in excluding particular users, and lack of clear management frameworks or standards [6].

Knowledge on effects of environmental noise on humans has been gained primarily from the field of noise abatement. Initiated by the Noise Act in 1972, the United States’ Environmental Protection Agency (EPA) defined thresholds of noise considered detrimental to health, and which are still used as guidelines for environmental noise management today [7]. Although the agency is no longer funded to enforce this mandate, The Noise Act led to an extensive body of work through the 1970s and early 1980s documenting the impacts of environmental noise on human hearing loss, health, and stress for a range of thresholds [8]. Much of this research, however, is based in the fields of occupational health or urban planning, with a focus on acute levels or specific types of noise (e.g., air or road traffic). While this provides an important foundation of knowledge, a key limitation is that management of environmental noise at larger scales depends on understanding the impacts not only of noise levels but of differing types and quality.

The detrimental effects of environmental noise on animals have been demonstrated across terrestrial, marine, and freshwater environments. These impacts are diverse, including physiological stress, behavioral avoidance, and interference with communication associated with mating, predator avoidance, and prey detection [9], [10]. Although it is widely recognized that increasing noise levels generally lead to greater effects on animals, recent research suggests that sound type, duration, and dominant frequency also play a significant role [11]. The range and complexity of responses creates a particular management challenge in that it is difficult to determine critical environmental noise thresholds except at species-specific levels. In general, mitigation options are also limited, and involve relatively heavy-handed approaches such as through recovery plans of species listed under the U.S. Endangered Species Act (e.g., [12]).

The unwieldy implications of mitigating for both human noise thresholds and wildlife-specific impacts have led some ecologists to adopt a more holistic approach through analysis and management of the *soundscape*. The term was first published in urban planning literature [13], and refers to all sounds – whether naturally occurring or human derived – that occur in a landscape. Descending from the field of landscape ecology, the nascent study of the characteristics and biological consequences of sounds within a landscape has recently been termed *soundscape ecology* [3]. By incorporating not only sound levels but sound type, patterns, and characteristics, soundscape ecology has application at large scales and in areas which may not suffer from acute noise pollution, but where the acoustic experience (for people and animals) is nonetheless important. A prime example is the National Parks system in the United States, where visitors rank noisiness as a primary determinant of the quality of their experience [14]. Acting under a dual mandate to provide recreational opportunities while conserving natural resources, the National Park Service has identified protection of natural sounds as a priority, and is conducting research in many parks toward understanding and managing environmental noise from a soundscape perspective [15]. Soundscape ecology also offers the potential to track changes in ecological status and integrity over time and space [16], such as monitoring the impacts of large-scale environmental processes like climate change or different types of development (e.g., [17]).

As soundscape ecology is still in its infancy, many research gaps exist which, as they are addressed, will move soundscape techniques from theoretical to practical application in managing environmental noise. Principal among these is that, while preservation of natural soundscapes is critically important, the majority of our time is spent in more mixed-use landscapes such as parks, residential, and commercial areas. As more landscapes are converted to agricultural and urban areas in the future, maintaining ecological integrity in human dominated systems will be a critical management challenge. Understanding soundscape dynamics and the potential for application to environmental noise management across types of development is therefore of fundamental importance [16].

Thus far few studies have described acoustic characteristics across a gradient of urbanization or as a function of land use (but see [18]), and to our knowledge, none have been conducted for freshwater lakes. In this study we measured and described sound characteristics for lakes in the Puget Sound lowlands of western Washington State. Land cover prior to European settlement was mixed evergreen and deciduous forest with scattered prairies, but sizeable portions of the Puget Sound lowlands have been converted to urban uses of varying intensities in association with cities like Seattle, Tacoma, and their suburbs [19]. This pattern of increasing urbanization is occurring within many regions of the United States [20]. Our objectives were 1) to quantify the spatial and temporal acoustic characteristics of lakes both above and below water, 2) describe and compare characteristics of biological and human-derived sounds across an urbanization gradient, and 3) test for human and environmental correlates of dominant acoustic patterns.

Our choice of study system (freshwater lakes) was motivated by multiple research priorities. As part of freshwater networks, lakes are valuable natural resources under considerable pressure from multiple uses, and represent sentinels to track changes in ecological integrity over time and space [21], [22]. Further, freshwater organisms are among the most imperiled worldwide and are facing the largest decreases in biodiversity due to combined threats of urbanization, climate change, and invasive species [23]. Recreational boaters can create substantial noise pollution [24], with documented impacts on fish [25] and waterfowl [26], and there is growing awareness of the need to study anthropogenic noise and its effects on aquatic ecosystems. Lastly, as highly valued residential and recreational areas, the acoustic environment (or soundscape) of lakes is often an important part of a resident or visitor’s experience. As such, lake systems are in particular need of advances in technology and tools to assist in monitoring and mitigation of environmental consequences of development [16]. A final consideration of our study is to advance the science of soundscape ecology and management by identifying acoustic metrics which have human and ecological relevance. These metrics would then be available to developers, urban planners, and communities for the purposes of self management of environmental noise or the setting of acoustic targets [6].

## Methods

### Site Selection and Recording

**Figure 1:**
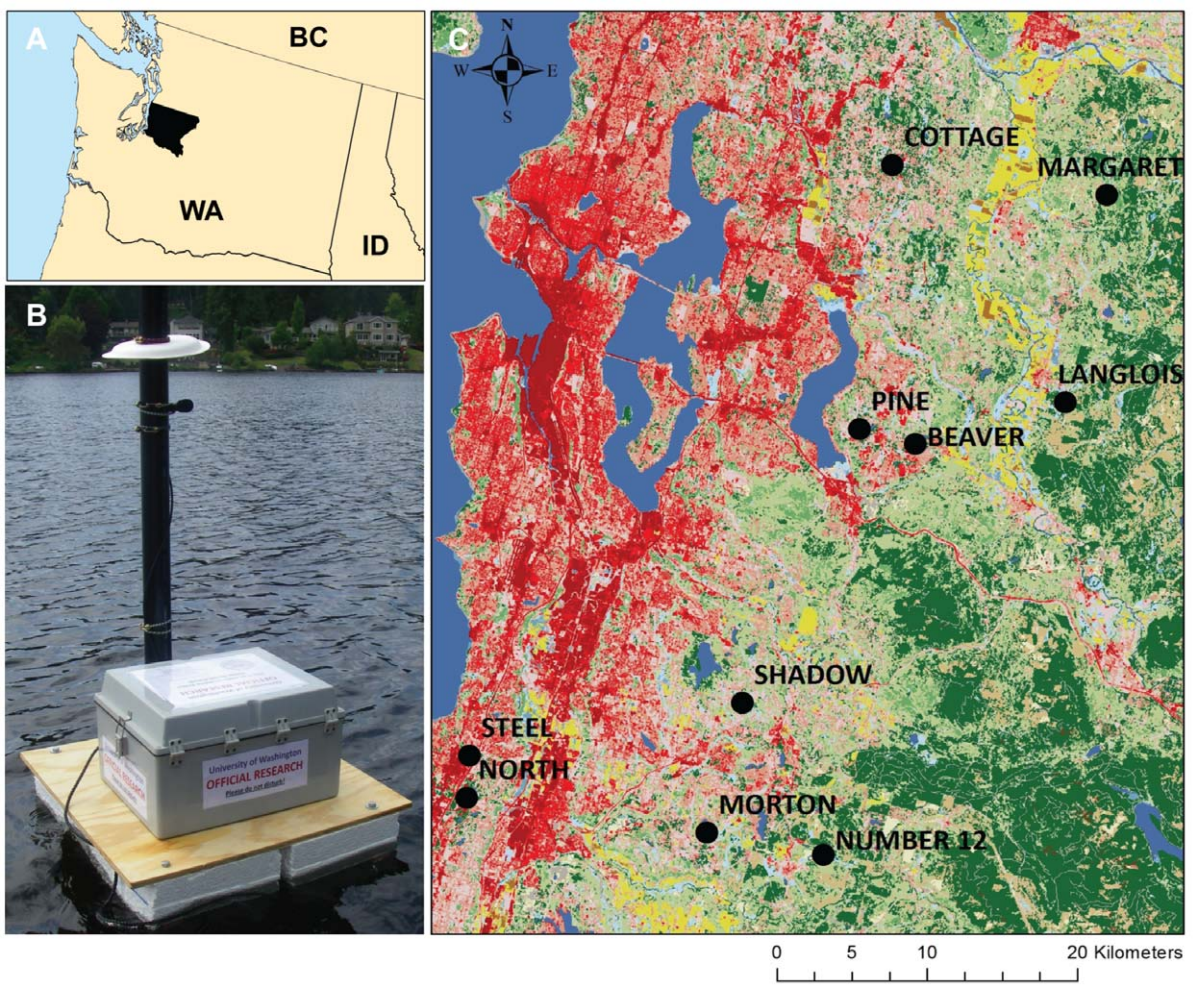
Study area, apparatus, and sampling locations. A) King County in Washington State, U.S.A., B) floating platform with microphone, and C) location of lakes across an urbanization gradient (green = forested; red = urbanized). The city of Seattle is located on the left (west) of the map.

We deployed a digital audio field recorder (SongMeter SM2, Wildlife Acoustics) on ten lakes in the Puget Sound lowlands of western Washington State (Fig. 1) between mid-June and late-July for a single 24-h period. Sampling during summer months allowed us to capture the influence of anthropogenic noise when outdoor activity is at a peak, and reflect the status of lake soundscapes during the season of greatest recreational value to park users and lake residents. The weatherproof recording unit was protected by enclosure in a hard plastic box mounted on a floating platform (75 cm *w*×75 cm *l*) which was anchored in the lake (Fig. 1); microphones were positioned from the anchored platform. Two-channel recording allowed simultaneous connection of omnidirectional hydro- and air microphones (Models: HTI-96 and SMXII) from the platform. The hydrophone was secured to a cinder block positioned on the lake bottom, and the air microphone attached at a height of 75 cm on a PVC pipe attached upward from the platform. The recorder was set to record sounds within 0–8 kHz, a frequency range which encompasses the majority of sounds generated by anthropogenic sources (*anthrophony*) and physical processes (*geophony*), as well as the large proportion of biological sounds generated by animals (*biophony*) [3], [27]. Sound was sampled at a rate of 44.1 kHz and 16 bits digitization; files were saved as raw uncompressed.*wav* files, and downloaded at the end of each individual lake sampling.

The study lakes were selected to be comparable in surface area (0.16–0.35 km^2^) and shoreline morphology (Table 1); we further restricted selection to those which prohibit motorized watercraft. Within these criteria, we chose lakes across a gradient (0–66%) of urban land cover quantified according to the percent of impervious surface within a 10 km radius of the center of the lake (source: USGS Land Cover Institute). For the purposes of some analyses we also categorized lakes as Low (<30%), Medium (30–50%), and High (>50%) urbanization (Table 1).

**Table 1:** Characteristics and dates of lakes sampled.

Lake Name	Sampling Date	Lake Area (km^2^)	Urban (%)	Category	Depth (m)	Public Park	Parcels (km-^2^)
Margaret	22-Jun-11	0.18	13	Low	13.1	No	566
Langlois	21-Jun-11	0.16	15	Low	29.9	No	44
Twelve	26-Jul-12	0.18	25	Low	8.5	No	416
Beaver	20-Jun-11	0.25	34	Med	16.5	Yes	407
Morton	16-Jun-11	0.28	40	Med	7.0	No	487
Pine	22-Jul-11	0.35	43	Med	11.9	Yes	434
Shadow	13-Jun-11	0.20	50	High	13.7	No	333
Cottage	23-Jun-11	0.25	52	High	7.6	Yes	314
Steel	17-Jun-11	0.19	62	High	7.3	Yes	543
North	25-Jul-12	0.23	66	High	10.4	Yes	353

We selected five lakes that contained a public park feature and five lakes without to examine differences in noise levels at lakes with greater public and recreational activity. Because parks are located disproportionately in areas of higher population density, this feature is represented more at the higher end of the urbanization gradient, but we included it as a measure of local-scale development which might influence noise levels. Residential development was a common feature at all lakes which might also influence acoustic characteristics; a measure of this local scale development was quantified according to the density of housing parcels by lake area (km^2^) based on the King County database (iMAP, King County GIS Center). All lakes contained foot-accessible boat launches.

Although weather patterns are fairly stable during the peak summer months in which we sampled, the effects of wind and rain were minimized by choosing 24-h periods during calm weather without precipitation and when winds were forecasted at less than 10 km•h^−1^; actual wind speeds measured during deployment and recovery were 3.6±2.4 km•h^−1^(mean maximum ± SD). The unit was deployed only on weekdays to minimize potentially dramatic differences in sound level due to increased use of public parks on weekends. The platform was set at a distance of 15 m from the shoreline to allow placement in 2–3 m of water, discouraging equipment interference by lake users. When a park was present, the platform was deployed in front of park shoreline; if no park was present, the platform was set in front of the boat launch or public access point to the lake. The hydrophone was secured at a depth of approximately 1.5 m (the mid-point of the water column), and the air microphone directed towards the shoreline. The majority of the lakes were sampled in 2011, but separate technical difficulties at two lakes resulted in sampling being done the following year (Table 1). The hydrophone was challenging to deploy overnight depending on vegetation and lake substrate (e.g., vulnerable to tipping over in soft sediment or sloped bottoms), resulting in a smaller number (*n* = 7) of successful site collections.

No specific permits were required for the described field sampling. All lakes were publicly accessible via boat launches maintained by either Washington State Department of Fish and Wildlife (WDFW) or King County Parks and Recreation. As we collected no biological samples, state scientific collection permits did not apply, and our sampling activities fell under and were in accordance with Public Conduct Rules for WDFW lands. Lake residents and users were notified by signage at the public park (if present), boat launch, and nearby residential docks.

### Data Analysis

Two approaches are used to describe and assess the acoustic characteristics of lakes across an urban gradient. First, we quantified two summary metrics representing the relative contributions of anthropogenic and biologically derived sources of sound. Univariate statistical methods are applied on these metrics to test the effect of landscape-scale factors on anthrophony and biophony. Second, we deploy multivariate statistical techniques on the entire sound spectrum (above-water samples) to describe differences in acoustic properties between lakes, and test the significance of landscape factors in defining those patterns. We end by comparing and contrasting the conclusions and strengths of each method.

### Sound Selections and Processing

From the recorded data for each lake, the 15 min at the top of each hour were extracted into separate.*wav* files; this process was conducted separately for air and hydrophone sound data. This resulted in a potential total of 240 above-water (10 lakes×24 hours) and 168 below-water (7 lakes×24 hours) sound files. Although the effects of rain and wind were minimized by deploying the apparatus during favorable weather, even wind speeds of 5 km•h^−1^ could potentially cause feedback; all selections were screened for feedback effects due to wind, and a different 15-min period during the same hour chosen if necessary.

To identify general temporal patterns in overall sound as well as the correspondence between above- and below-water sound, the power within each hourly sound selection was determined using the average power measurement in Raven Pro (version 1.4). Raven calculates this metric (in dB) as the sum of the power spectral density divided by the number of time-frequency bins in the selection. An important note is that Songmeter recorders are designed to capture unmanipulated sound by recording with a flat frequency response from 0.20 to 20 kHz. This contrasts with a large majority of environmental noise studies that record sound using common weightings (e.g., A or C), and generally report findings in dBA, a scale considered most relevant to human perception of sound. Although our data processing results in measures of power in dB (not dBA), we use these metrics in relative terms, and (with one exception) do not attempt to compare our results to studies related to specific thresholds of human hearing or health.

To create acoustic components which describe relative contributions of anthropogenic and biological noise, the acoustic intensity in each of eight 1-kHz intervals were calculated using the same average power measurement. We chose to quantify 1-kHz intervals to facilitate comparison of our results with prior works [18], [27], [28], which have also established the utility of these frequency bands in describing dominant soundscape characteristics [29]. The resulting matrix was row standardized across frequency intervals to generate a second matrix of the relative power in each frequency to the total sound. From these estimates of power across frequency intervals, we summarized metrics for frequencies which have been shown to strongly represent biophony and anthrophony. Anthrophony, which is commonly driven by factors such as air traffic, proximity to roads, or construction, occurs across all frequencies but is concentrated below 3 kHz [30], [31]. Because the lowest frequency range (0–1 kHz) is particularly vulnerable to variation from wind feedback, we selected the power of the central 1–2 kHz frequency interval as our conservative measure of anthrophony [18].

Biophony, representing the biological sounds of organisms in a landscape, generally occurs within the frequencies of 2 and 8 kHz [3]. A challenge in the analysis of biophony (or structure of higher frequency sounds) in developed environments is the potential for false positives created by background noise, which is highly typical of anthropogenic settings [28]. We addressed this issue by first excluding frequency intervals below 3 kHz. For each time selection, the maximum standardized (i.e., relative) power between the 3 and 8 kHz frequency intervals was selected to represent biophony. The methods we describe of filtering low frequency sounds and standardizing against a measure of total amplitude has been used effectively in previous studies [18], [28] which also isolated components of biophony for analysis.

### Statistical Analysis

To examine temporal trends in anthropogenic noise by urbanization level, we plotted the average power in the 1–2 kHz frequency interval (anthrophony) by hour of the day and urban category (Low, Medium, High) in which the sound sample was collected. We selected the 1–2 kHz interval as it strongly represents levels of human-generated sound [18] and is also comparable to A-weighted (dBA) sound, which is minimally weighted (+0–1.2 dB) between 1–2 kHz [32]. The similarity to dBA allows comparison with studies, such as those done by the Environmental Protection Agency, which established human noise thresholds for “outdoor annoyance and disturbance” [7].

Correlation between above- and below-water sound was tested by linear regression on average power of the total sound sample, summarized by lake and microphone type. We further examined whether particular frequencies were more strongly associated by regression on the power of individual frequency bands above and below water.

To identify spatial and temporal factors driving soundscape characteristics, we tested the importance of landscape factors and time period on anthrophony and biophony (hereafter based on above-water recordings) using multiple linear regression analysis. Based on prior studies, we expected anthrophony and biophony to vary independently according to general time periods (e.g., quietest at nighttimes, greater biophony in dawn or dusk periods) [28], [33]. We therefore summarized response variables according to four categorical time periods: Night (22∶00–03∶00), Morning (04∶00–09∶00), Day (10∶00–15∶00), and Evening (16∶00–21∶00). The full regression model included percent urbanization, parcel density, park presence, and time period as fixed effects. We initially included interaction terms for urbanization and time period, but removed these from the final model as there were no significant interactions or improvement in model fit (based on adjusted R^2^).

We next explored the potential of multivariate approaches to describe acoustic characteristics of sites and test significance of the same fixed effects (e.g., urbanization, time period) in driving patterns in variation. A primary benefit of multivariate methods in analysis of frequency intervals is the potential to identify dominant gradients in variables expected to demonstrate some degree of correlation, while taking advantage of the additional information provided by the larger suite of information. We used principal component analysis (PCA) to summarize patterns of multivariate variation using the standardized matrix of average power for all eight frequency intervals. The PCA was paired with permutational tests of significance (PERMANOVA on standardized data with 9,999 permutations; [34]) and tests of homogeneity of multivariate dispersions (PERMDISP on standardized data with 9,999 permutations; [35]) between factors of urban level, park presence, and time period.

PERMANOVA tests for significant differences of factor levels (e.g. Low/Medium/High urban) in position (central tendency) of the multivariate centroid for each factor, and PERMDISP tests for differences in dispersion (variability) around the centroid within factor levels. Although permutational approaches offer greater freedom from assumptions of normality and heteroscedascity, the multivariate data set was nonetheless examined for and met assumptions of univariate and multivariate normality. Unlike the univariate regression analysis, we did not include parcel density as a factor, as PERMANOVA and PERMDISP require categorical variables. All statistical analyses were done in the R Programming Environment (The R Project for Statistical Computing, http://www.r-project.org/); multivariate analyses were conducted using the vegan package [36].

## Results

**Figure 2:**
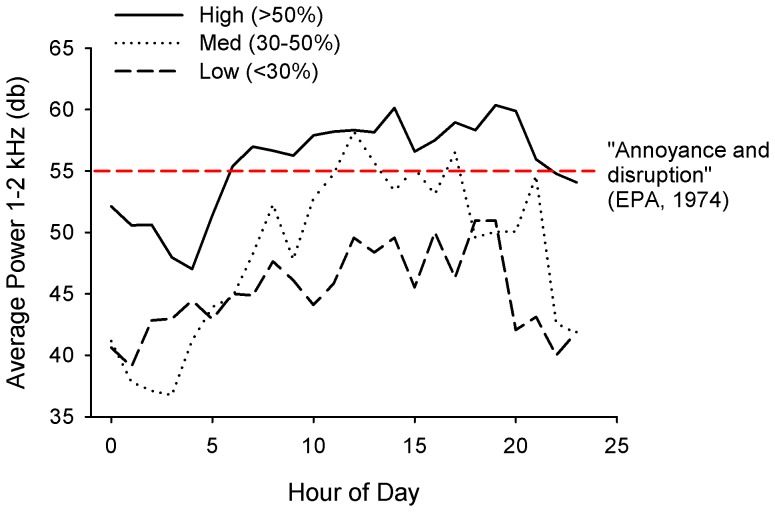
Temporal trends in anthropogenic sound by urban category. Average power (1–2 kHz) at lakes characterized as Low (<30%, *n* = 3), Medium (30–50%, *n* = 3), and High (>50%, *n* = 4) urbanization. The threshold established by the U.S. Environmental Protection Agency (in dBA) for “outdoor annoyance and disruption” [7] is shown as a reference; dBA is highly comparable in this frequency range, with a weighting value of +0–1.2 dB between 1–2 kHz; we have considered these equivalent for purposes of illustration.

Anthropogenic noise was significantly and positively correlated with urbanization (Pearson’s, *R^2^ = *0.55, *p = *0.01), with distinct differences in temporal patterns between High, Medium, and Low urban categories (Fig. 2). All lakes were quietest at night, increasing in noise levels over the course of the day. The most urbanized lakes increased sharply and early in power and remained high throughout the day, while low urbanization in the surrounding area resulted in a gradual rise and peak in the evening. Moderately urbanized lakes demonstrated an intermediate pattern. Greater levels of anthropogenic sound were consistently associated with higher urbanization at all hours; the percent of hourly measurements which exceeded 55 dB was 67%, 17%, and 0%, respectively, for lakes characterized as High, Medium, and Low urbanization. The U.S. Environmental Protection Agency defines this threshold for “outdoor annoyance and disruption” [7].

**Figure 3:**
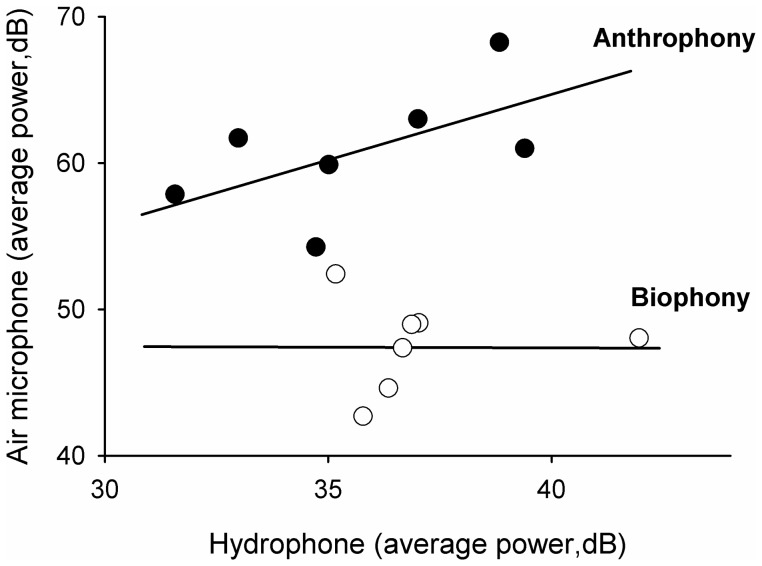
Relationship of above- and below-water sound. Patterns and correlation of overall sound (average power in dB) between above-water and hydrophone data by frequencies corresponding to anthrophony (•, mean: 1–3 kHz) and biophony (Ο, mean: 3–8 kHz). Regression on individual frequency intervals resulted in a significant correlation for only the 0–1 kHz band (*R^2^* = 0.71, *p* = 0.02, all others *R^2^*<0.03, *p*>0.69).

There was strong correspondence between the average power of above- and below-water sound across lakes (Pearson’s, *R^2^ = *0.71, *p = *0.06*)*. Regression of individual frequency intervals between above- and below-water sound resulted in only one significant relationship between 0–1 kHz (*R^2^* = 0.71, *p* = 0.02, all others *R^2^*<0.03, *p*>0.69), suggesting that the overall correspondence was primarily driven by low frequency sounds (likely derived from human activities) permeating the water (Fig. 3).

Multiple regression analysis resulted in differing patterns of importance of landscape and temporal factors on the relative power of anthrophony and biophony (Table 2). Urbanization positively contributed to anthrophony (Table 2), and was the only significant factor in the regression model (*R^2^* = 0.51, *F*_6,33_ = 7.79, *p*<0.001). Regression modeling for biophony (*R^2^* = 0.46, *F*_6,33_ = 6.46, *p*<0.001) indicated that urbanization contributed negatively to levels of biophony, but this relationship was not significant (Table 2). Instead, presence of a public park had a significant negative effect on biophony. Biophony was significantly greater in all time periods, with the largest and most significant increase in the Morning period.

**Table 2:** Regression models for fixed effects of spatial and temporal factors on relative power of anthrophony and biophony.

	*β*	*SE*	*T*	*p*
**Anthrophony**				
*Intercept*	0.123	0.006	22.186	<2e–16
Urban (%)	0.033	0.011	3.139	**0.004**
Public Park	0.006	0.004	1.681	0.102
Parcel Density (km^−2^)	0.000	0.000	0.032	0.974
Morning	−0.006	0.004	−1.394	0.173
Day	0.001	0.004	0.223	0.825
Evening	0.003	0.004	0.688	0.496
**Biophony**				
*Intercept*	0.115	0.002	48.837	<2e–16
Urban (%)	−0.005	0.005	−1.115	0.273
Public Park	−0.004	0.002	−2.792	**0.009**
Parcel Density (km^−2^)	0.000	0.000	0.230	0.819
Morning	0.009	0.002	5.438	**0.000**
Day	0.007	0.002	3.916	**0.000**
Evening	0.007	0.002	3.871	**0.000**

Regression was on mean power by time period for each lake (*n* = 40); significant fixed effects (*p*<0.05) are highlighted in bold. Tests for interactions of fixed effects resulted in no significant relationships so interaction terms were removed.

**Figure 4:**
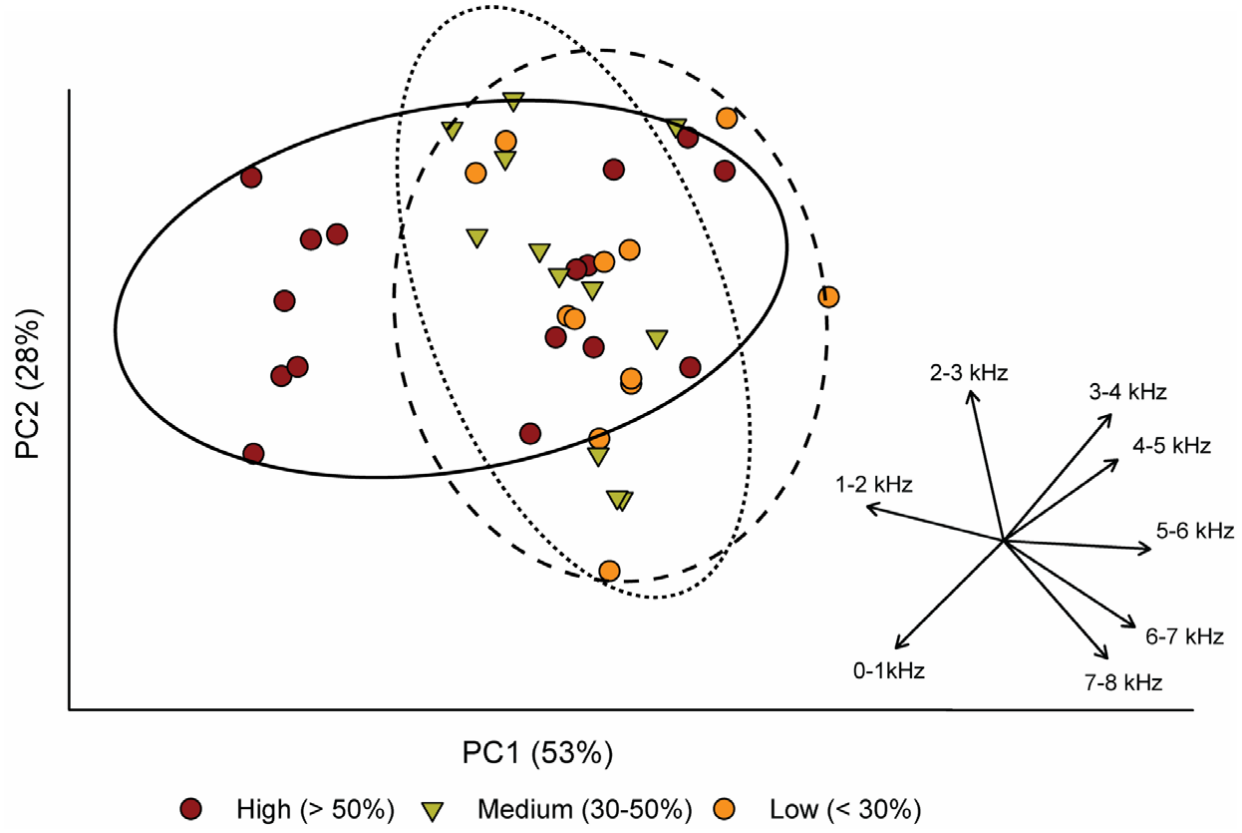
Dominant frequencies and acoustic variation by urban category in multivariate space. Principal component analysis summarizing patterns of relative power in eight frequency intervals across the study lakes. Each data point represents one of four time periods at an individual lake. Urban categories are delineated with ordination hulls (90% confidence interval) according to High (solid), Medium (dotted), and Low (dashed) surrounding urbanization. Inset displays the component loadings (eigenvectors) for each frequency interval.

Principal component analysis of the relative power in all eight frequency bands (performed for above-water sound only) identified dominant gradients which corresponded well with expected patterns of anthrophony and biophony. The first principal component (PC1) explained 53% of the variation (*p*<0.001) and primarily distinguished power in the 1–2 kHz and 5–6 kHz frequency intervals, which were inversely correlated with each other (Fig. 4). This indicates that the largest portion of variation in the dataset was determined by low frequency, anthropogenic power, and a corresponding negative relationship with a higher frequency associated with biological sources. The 2–3 and 3–4 kHz frequency intervals were most strongly associated with principal component axis 2 (PC2), which explained a lower but still significant (28%, *p*<0.001) amount of variation in the soundscape dataset. The relationship of increasing urbanization with anthropogenic sound is reflected in the ordination plot (Fig. 4) which shows the largest dispersion along PC1 in the High urban category, while lakes characterized as Medium and Low are more similar in their acoustic characteristics, and located toward greater relative power in higher frequency intervals in multivariate space. Together, PC1 and PC2 explained 81% of the variation in the soundscape dataset.

According to multivariate testing for significant effects of landscape and temporal factors (PERMANOVA and PERMDISP) on acoustic patterns, significant differences in overall position of the centroid existed within all model factors: urban category (*Pseudo-p* = 0.004), presence of a park (*Pseudo-p* = 0.007), and time period (*Pseudo-p* = 0.010). Differences in dispersion (variability) of acoustic power between factor levels were more minimal, with urban category as the only factor tested which showed significant differences in dispersion around the centroid (permuted *p* = 0.04; Fig. 4).

**Figure 5:**
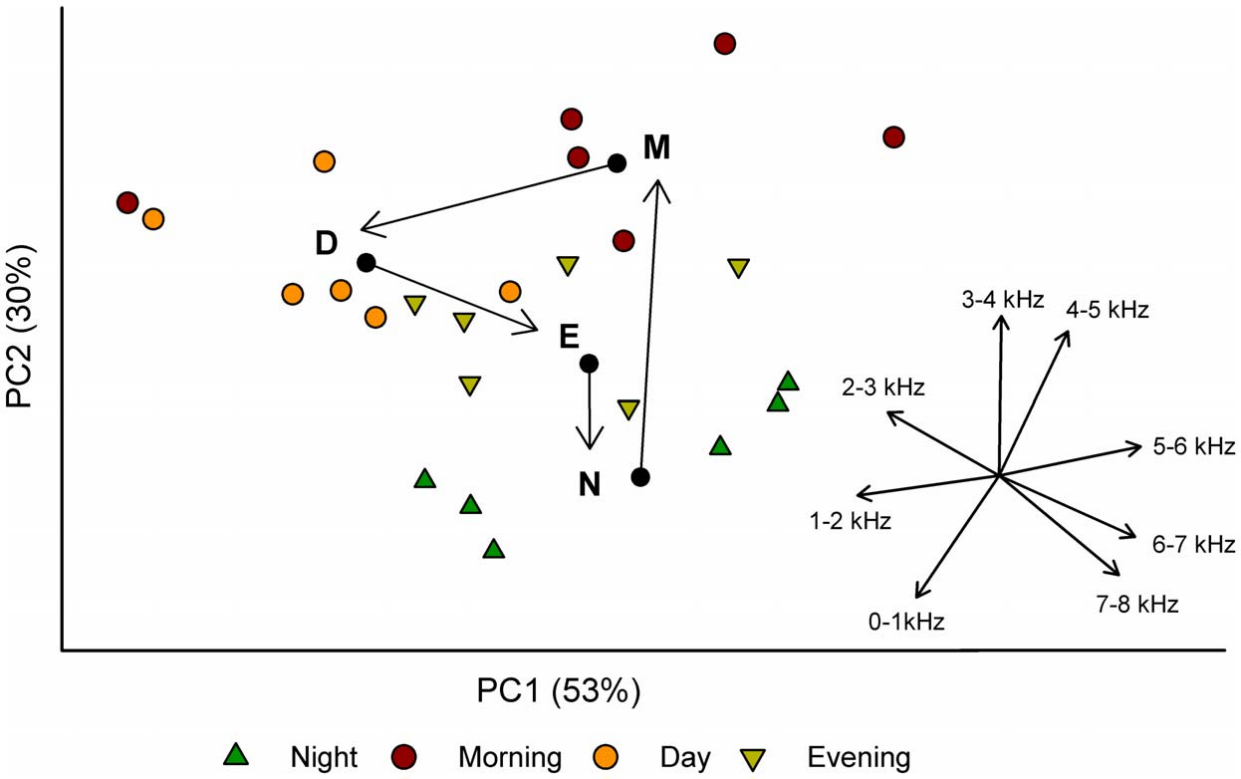
Changes in importance of frequency intervals by time period for a single lake. Principal component analysis summarizing patterns of relative power in eight frequency intervals using all 24 time selections for Morton Lake (urbanization = 40%). Labels delineate the multivariate centroid for individual data points for each time period: Night (N), Morning (M), Day (D), and Evening (E). Inset displays the component loadings (eigenvectors) for each frequency interval.

For the purposes of demonstrating potential application of these multivariate approaches to monitoring and managing soundscapes, we conducted a second PCA on all 24 (hourly) sound selections for a single lake (Morton Lake, urbanization = 40%). The resulting ordination plot (Fig. 5) shows changes in relative acoustic power of the eight frequency bands over the four different time periods, with a shift to greater power in higher frequency intervals in the morning and then to increasing proportions of low frequency anthrophony during the day. Evening is characterized by a reduction in anthrophony and some increase in higher frequency sounds.

## Discussion

The conversion of landscapes to mixed-use, semi- and fully urbanized settings is creating new dilemmas around quality of life for humans and animals. Often, we want the convenience of development while reducing collateral damages such as pollution, reduced biodiversity, and noise. In other cases, the very nature of common-pool resources (i.e., water, air, or the soundscape) means that single users or types of use have disproportionate impacts on others, but exclusion may be unfeasible, unenforceable, or even unethical [37]. Within this context, communities, planners, and managers must develop new frameworks and methods to determine priorities and balance competing interests [6]. In this study, we describe the effects of large- and local-scale development on the soundscape of recreational and residential lakes in western Washington State, U.S.A., and illustrate use of multivariate methods in the analysis of soundscapes for practical management purposes.

The temporal and spatial patterns we identify build upon those documented in other studies examining sound levels as a function of urbanization. Although many studies have focused on noise effects from specific point sources (e.g., airports or highways), fewer have tested sound in terms of landscape metrics with relevance at more general ecological scales. One of the first efforts to quantify this relationship was an EPA study [38] which successfully modeled A-weighted sound levels over a 24-hour period as a function of population density; this represents a useful model but was intentionally designed to focus on urban residential areas. More similar to our approach, Joo et al. (2011) used satellite-derived, urban land cover data to assess landscape patterns in anthro- and biophony. They found significant increases in anthrophony with some land use types (agricultural and commercial), and with some (40–60%), but not all levels of increasing urbanization. Our results correspond strikingly, however, with those of Warren et al. (2006), who found a similar significant correlation in sound pressure levels against distance from an urban center reflecting lower landscape urbanization (Phoenix, AZ; *R^2^* = 0.46) as compared with our metric of percent urbanization (*R^2^* = 0.55). Overall, we found that noise levels, particularly from anthropogenic sources, are predictable across large spatial scales, supporting recent efforts to map noise propagation using Geographic Information Systems [39]. The temporal patterns that we identify of increases in anthropogenic noise during the day, and significantly greater biophony associated with dawn periods (the “dawn chorus”) are also consistent with other studies [28], [33]. We found more limited support for increased biophony at dusk; although a weaker biophony signal at dusk is consistent with previous work [27], [28], our results indicate that increased anthropogenic noise during day and evening is also more likely to interfere with analysis and interpretation of biophony during these time periods.

The effect of urbanization on sound level and type creates considerable ecological challenges in that natural sounds are masked or obscured by a wide variety of human-related sounds. Urban lakes, particularly those with public park features, had proportionally lower biophony compared to lakes in less urbanized landscapes. This phenomenon may be a result of reduced species diversity in urban parks as compared to natural areas [40], [41], and would be consistent with Joo et al. (2011), who found a positive correlation of biophony with diversity of bird species present, and a corresponding negative correlation with anthrophony. An important consideration in the interpretation of biophony in urbanized settings, however, is the problem in distinguishing sources of higher frequency sounds. A variety of high frequency anthropogenic sounds (e.g., voices, power tools, dogs barking) as well as collateral noise from low frequency sources can result in misclassification or false positives to biophony [28]. Our results suggest that this difficulty becomes greater as anthrophony increases, potentially complicating the use of acoustic surveys for species diversity [17] and for analysis and management of soundscapes in developing areas [16]. We suggest, however, that identifiable patterns across an urbanization gradient may allow for development of correction factors in analysis, guide experimental designs [33], and also indicate thresholds for testing noise impacts on particular species of concern (e.g., masking, change in acoustic niche), as well as community composition and ecosystem processes [10], [42].

A surprising finding of our study is the urbanization signature in the underwater recordings. By designing our study to exclude the influence of underwater sounds from motorized boats, which have been relatively well studied [24], [43], we identify a less well known phenomenon of underlying urban noise pollution which may affect aquatic organisms. While the magnitude is less than noise from boat engines, the effect is likely to be more consistent, suggesting that the biological implications for fish, amphibians, and reptiles are ripe for investigation. This study restriction, however, means our above-water results are likely to be conservative; ecologists seeking to use these as a benchmark should consider that lakes which allow motorized watercraft will have much higher levels of anthropogenic noise.

Along with potential impacts to wildlife, our findings describe the negative acoustic trends in commonly used environments, or what Dumyahn and Pijanowski (2011) refer to as “everyday soundscapes” [16]. Millions of people use public parks to satisfy their need for recreation and to provide opportunities to connect with nature. Although recent evidence indicates that nature-based recreation on national public lands may be declining [44], visitation of natural areas near urban centers are only likely to increase due to travel limitations imposed by rising oil costs and a declining economy [45]. As the primary place that people access nature, local parks and preserves shape economic growth and residential development patterns [46], and are associated with a range of human health benefits [47]. Although local parks are used for somewhat different reasons compared to National Parks, we believe that lessons can be learned from the latter, where intrusive sounds are a matter of concern to visitors. As was reported to the U.S. Congress in the “Report on the Effects of Aircraft Overflights on the National Park System” (1995) a system-wide survey revealed that equal numbers of visitors come to national parks to enjoy the natural soundscape as to view the scenery (91% and 93%, respectively). Our study indicates that noise pollution can be substantial on lakes surrounded by urbanized lands, where public parks are commonly located, and that current sound levels may compromise the intended health and ecosystem benefits of these areas. Our results further show that large-scale patterns of urbanization are more important than local development in determining the soundscape. This suggests two potential courses of action in 1) acoustic monitoring of recreational areas to achieve visitor experience and health-related outcomes, and 2) promoting development of some parks in less urbanized areas to serve as not only recreational but acoustic oases.

Our study findings also illustrate use of multivariate approaches in analysis of soundscapes which we believe have important implications for moving soundscapes from theoretical to practical management application [3]. Principal component analysis explained a large majority of variation in the acoustic data and described similar temporal and spatial patterns as regression analysis. The strength in the multivariate approach, however, is in independence from *a priori* determination of significant acoustic components, which also substantially simplifies data processing and analysis. Given that a fundamental challenge in acoustic sampling is to minimize the substantial effort of collecting and processing large sound files while choosing samples which adequately represent and characterize the acoustic environment, this benefit is not trivial. In our results, we present changes in acoustic patterns for a single lake over time (24 hours), but this method could prove equally useful to track changes in the soundscape across other factors such as season (e.g. spring mating times for birds, or periods of increased human use), land use type, or environmental conditions (e.g., sunny as opposed to cloudy weather). Furthermore, although only briefly explored in our study, information on multivariate dispersion or acoustic diversity can also be used to compare differences between locations or factors of interest. Overall, we believe these methods are highly useful in identifying dominant frequencies and tracking changes within locales, and may contribute to an identified need for soundscape management and conservation tools based not only on sound levels but sound quality [16]. For example, communities, managers, or developers could set (and effectively monitor) acoustic targets for an area (e.g., reduce levels of anthropogenic noise during particular times of day, or increase proportion of biophony) and create noise abatement programs or develop landscapes accordingly.

Although we believe this work is a substantial contribution to the field of soundscape research, there are considerations that should accompany interpretation of these results, as well as be used to guide future research, particularly in urban or semi-urban areas. Our study is based on a single day of sampling at each site, despite the fact that low cost, multi-day recording is not only possible, but often a primary benefit of acoustic monitoring. When deployed at heavily utilized urban lakes, the recording equipment was highly vulnerable to accident or vandalism (e.g., a preliminary sampling attempt failed when a swimmer used the platform as a personal flotation device). We therefore sought to minimize deployment time, but chose a longer sampling unit of 15 minutes (at 24 time points) to gain a representative sound sample. As a point of contrast, a prior study which also examined sound across an urban gradient [18] used recordings from a longer period (two days) each month but chose a smaller sampling unit (3 minutes at 6 time points). Additionally, our focus was to characterize the influence of anthropogenic noise across an urbanization gradient. We expected that sources of this noise (e.g., air and road traffic, park and residential use) would not vary substantially from one weekday to another; our sound samples were also screened for unusual events. We believe that this sampling design resulted in a good representation of large-scale patterns in anthropogenic noise associated with urbanization, but use of multi-day recordings would offer more nuanced descriptions of lake soundscapes. Future research which incorporates multi-day sampling would also offer opportunities to investigate the effects of season, as well as differences in weekday and weekend noise patterns. These factors were intentionally excluded from our study, but might be of considerable importance to developers or urban planners.

The potential for the new field of soundscape ecology to monitor and mitigate negative effects of urbanization depends greatly on understanding general patterns as well as development of relevant metrics and methods of analysis [3], [29]. In this study, we contribute to these goals by identifying sound levels and characteristics associated with development in and around a large metropolitan area, and relating these to current understanding of noise impacts on humans and wildlife. Overall, our findings emphasize the importance of the urban gradient in structuring the acoustic environment, and the need for ecological studies which incorporate or account for these trends. Further, we offer a relatively simple method of analysis and monitoring of soundscapes over time and space, and recommend future research which tests robustness of the approach in actual management scenarios.

## References

[1] Vitousek PM (1997) Human domination of earth’s ecosystems. Science 277: 494–499 doi:10.1126/science.277.5325.494.

[2] Ellis EC (2011) Anthropogenic transformation of the terrestrial biosphere. Philos T Roy Soc A 369: 1010–1035 doi:10.1098/rsta.2010.0331. 10.1098/rsta.2010.0331 21282158

[3] Pijanowski BC, Villanueva-Rivera LJ, Dumyahn SL, Farina A, Krause BL, et al (2011) Soundscape ecology: the science of sound in the landscape. BioScience 61: 203–216 doi:10.1525/bio.2011.61.3.6.

[4] Hempton G, Grossmann J (2009) One square inch of silence: one man’s search for natural silence in a noisy world. New York: Free Press. 356 p.

[5] Barber JR, Burdett CL, Reed SE, Warner KA, Formichella C, et al (2011) Anthropogenic noise exposure in protected natural areas: estimating the scale of ecological consequences. Landscape Ecol 26: 1281–1295 doi:10.1007/s10980-011-9646-7.

[6] Dumyahn SL, Pijanowski BC (2011) Beyond noise mitigation: managing soundscapes as common-pool resources. Landscape Ecol 26: 1311–1326 doi:10.1007/s10980-011-9637-8.

[7] Environmental Protection Agency (1974) Information on levels of environmental noise requisite to protect public health and welfare with an adequate margin of safety. Washington, D.C.: U.S. Environmental Protection Agency, Office of Noise Abatement and Control. 150 p.

[8] Environmental Protection Agency (1980) EPA noise control program - progress to date, April 1980. Washington, D.C.: U.S. Environmental Protection Agency, Office of Noise Abatement and Control. 43 p.

[9] Barber JR, Crooks KR, Fristrup KM (2010) The costs of chronic noise exposure for terrestrial organisms. Trends Ecol Evol 25: 180–189 doi:10.1016/j.tree.2009.08.002. 19762112 10.1016/j.tree.2009.08.002

[10] Francis CD, Kleist NJ, Ortega CP, Cruz A (2012) Noise pollution alters ecological services: enhanced pollination and disrupted seed dispersal. P Roy Soc B-Biol Sci 279: 2727–2735 doi:10.1098/rspb.2012.0230. 10.1098/rspb.2012.0230 PMC3367785 22438504

[11] Kight CR, Swaddle JP (2011) How and why environmental noise impacts animals: an integrative, mechanistic review. Ecol Lett 14: 1052–1061 doi:10.1111/j.1461-0248.2011.01664.x. 21806743 10.1111/j.1461-0248.2011.01664.x

[12] National Marine Fisheries Service (2008) Recovery plan for southern resident killer whales *(Orcinus orca)* Seattle, WA: National Marine Fisheries Service, Northwest Region. Available: http://purl.fdlp.gov/GPO/gpo24027. Accessed 1 September 2012.

[13] Southworth M (1969) The sonic environment of cities. Environ Behav 1: 49–70 doi:10.1177/001391656900100104.

[14] National Park Service (2000) Director’s order No. 47: Soundscape preservation and noise management. Washington, D.C.: U.S. Department of Interior. Available: http://www.nps.gov/policy/DOrders/DOrder47.html. Accessed 10 August 2012.

[15] Miller NP (2008) US National Parks and management of park soundscapes: a review. Appl Acoust 69: 77–92 doi:10.1016/j.apacoust.2007.04.008.

[16] Dumyahn SL, Pijanowski BC (2011) Soundscape conservation. Landscape Ecol 26: 1327–1344 doi:10.1007/s10980-011-9635-x.

[17] Sueur J, Pavoine S, Hamerlynck O, Duvail S (2008) Rapid acoustic survey for biodiversity appraisal. PLoS ONE 3: e4065 doi:10.1371/journal.pone.0004065. 19115006 10.1371/journal.pone.0004065 PMC2605254

[18] Joo W, Gage SH, Kasten EP (2011) Analysis and interpretation of variability in soundscapes along an urban–rural gradient. Landscape Urban Plan 103: 259–276 doi:10.1016/j.landurbplan.2011.08.001.

[19] Alberti M, Booth D, Hill K, Coburn B, Avolio C, et al (2007) The impact of urban patterns on aquatic ecosystems: an empirical analysis in Puget lowland sub-basins. Landscape Urban Plan 80: 345–361 doi:10.1016/j.landurbplan.2006.08.001.

[20] Theobald DM (2010) Estimating natural landscape changes from 1992 to 2030 in the conterminous US. Landscape Ecol 25: 999–1011 doi:10.1007/s10980-010-9484-z.

[21] Carpenter SR, Benson BJ, Biggs R, Chipman JW, Foley JA, et al (2007) Understanding regional change: a comparison of two lake districts. BioScience 57: 323–335 doi:10.1641/B570407.

[22] Schindler DW (2009) Lakes as sentinels and integrators for the effects of climate change on watersheds, airsheds, and landscapes. Limnol Oceanogr 54: 2349–2358 doi:10.4319/lo.2009.54.6_part_2.2349.

[23] Dudgeon D, Arthington AH, Gessner MO, Kawabata Z-I, Knowler DJ, et al (2005) Freshwater biodiversity: importance, threats, status and conservation challenges. Biol Rev 81: 163–182 doi:10.1017/S1464793105006950. 16336747 10.1017/S1464793105006950

[24] Mosisch TD, Arthington AH (1998) The impacts of power boating and water skiing on lakes and reservoirs. Lakes & Reserv: Res Manage 3: 1–17 doi:10.1111/j.1440–1770.1998.tb00028.x.

[25] Popper AN, Fewtrell J, Smith ME, McCauley RD (2003) Anthropogenic sound: effects on the behavior and physiology of fishes. Mar Technol Soc J 37: 35–40 doi:10.4031/002533203787537050.

[26] York D (1994) Recreational-boating disturbances of natural communities and wildlife: an annotated bibliography. Washington, D.C.: U.S. Department of Interior, National Biological Survey. 30 p.

[27] Krause B, Gage SH, Joo W (2011) Measuring and interpreting the temporal variability in the soundscape at four places in Sequoia National Park. Landscape Ecol 26: 1247–1256 doi:10.1007/s10980-011-9639-6.

[28] Depraetere M, Pavoine S, Jiguet F, Gasc A, Duvail S, et al (2012) Monitoring animal diversity using acoustic indices: implementation in a temperate woodland. Ecol Indic 13: 46–54 doi:10.1016/j.ecolind.2011.05.006.

[29] Villanueva-Rivera LJ, Pijanowski BC, Doucette J, Pekin B (2011) A primer of acoustic analysis for landscape ecologists. Landscape Ecol 26: 1233–1246 doi:10.1007/s10980-011-9636-9.

[30] Cornillon PC (1977) Simple model for simulating traffic noise spectra. J Acoust Soc Am 61: 739–743 doi:10.1121/1.381362.

[31] Halfwerk W, Holleman LJM, Lessells CkM, Slabbekoorn H (2011) Negative impact of traffic noise on avian reproductive success. J Appl Ecol 48: 210–219 doi:10.1111/j.1365–2664.2010.01914.x.

[32] International Electrotechnical Commission (2001) IEC 60651 - Consolidated Edition 1.2 (incl. am1+am2). Geneva, Switzerland: IEC.

[33] Warren PS, Katti M, Ermann M, Brazel A (2006) Urban bioacoustics: it’s not just noise. Anim Behav 71: 491–502 doi:10.1016/j.anbehav.2005.07.014.

[34] Anderson MJ (2001) Permutation tests for univariate or multivariate analysis of variance and regression. Can J Fish Aquat Sci 58: 626–639 doi:10.1139/f01–004.

[35] Anderson MJ (2006) Distance-based tests for homogeneity of multivariate dispersions. Biometrics 62: 245–253 doi:10.1111/j.1541-0420.2005.00440.x. 16542252 10.1111/j.1541-0420.2005.00440.x

[36] Oksanen J, Blanchet FG, Kindt R, Legendre P, Minchin PR, et al. (2012) vegan: Community Ecology Package. Available: http://CRAN.R-project.org/package=vegan.

[37] Ostrom E (1999) Revisiting the commons: local lessons, global challenges. Science 284: 278–282 doi:10.1126/science.284.5412.278. 10195886 10.1126/science.284.5412.278

[38] Galloway WJ, Eldred KM, Simpson MA (1974) Population distribution of the United States as a function of outdoor noise level. Washington D.C.: U.S. Environmental Protection Agency, Office of Noise Abatement and Control. 71 p.

[39] Reed SE, Boggs JL, Mann JP (2012) A GIS tool for modeling anthropogenic noise propagation in natural ecosystems. Environ Modell Softw 37: 1–5 doi:10.1016/j.envsoft.2012.04.012.

[40] Melles S, Glenn S, Martin K (2003) Urban bird diversity and landscape complexity: species – environment associations along a multiscale habitat gradient. Conservation Ecology 7: 5. Available http://www.consecol.org/vol7/iss1/art5. Accessed 22 July 2012.

[41] McKinney ML (2008) Effects of urbanization on species richness: a review of plants and animals. Urban Ecosyst 11: 161–176 doi:10.1007/s11252-007-0045-4.

[42] Francis CD, Ortega CP, Cruz A (2011) Noise pollution filters bird communities based on vocal frequency. PLoS ONE 6: e27052 doi:10.1371/journal.pone.0027052. 22096517 10.1371/journal.pone.0027052 PMC3212537

[43] Slabbekoorn H, Bouton N, Van Opzeeland I, Coers A, Ten Cate C, et al (2010) A noisy spring: the impact of globally rising underwater sound levels on fish. Trends Ecol Evol 25: 419–427 doi:10.1016/j.tree.2010.04.005. 20483503 10.1016/j.tree.2010.04.005

[44] Pergams ORW, Zaradic PA (2008) Evidence for a fundamental and pervasive shift away from nature-based recreation. P Natl Acad Sci USA 105: 2295–2300 doi:10.1073/pnas.0709893105. 10.1073/pnas.0709893105 PMC2268130 18250312

[45] Beatley T (2011) Biophilic cities: integrating nature into urban design and planning. Washington, DC: Island Press. 191 p.

[46] Hansen AJ, Rasker R, Maxwell B, Rotella JJ, Johnson JD, et al (2002) Ecological causes and consequences of demographic change in the New West. BioScience 52: 151–162 doi:10.1641/0006–3568(2002)052[0151:ECACOD]2.0.CO;2.

[47] Frumkin H (2001) Beyond toxicity: human health and the natural environment. Am J Prev Med 20: 234–240 doi:10.1016/S0749-3797(00)00317-2. 11275453 10.1016/s0749-3797(00)00317-2

